# Making sense of “buzzword” as a term through co-occurrences analysis

**DOI:** 10.1016/j.heliyon.2021.e07208

**Published:** 2021-06-04

**Authors:** Elena N. Malyuga, Wayne Rimmer

**Affiliations:** Peoples' Friendship University of Russia (RUDN University), 6 Miklukho-Maklay Str., Moscow, Russian Federation

**Keywords:** Buzzword, Vogue word, Google Scholar, Co-occurrence, Title

## Abstract

The study argues that while the existing research on buzzwords mainly involves the functional and pragmatic analysis of their individual specimen, such as “engagement”, “synergy” or “development”, an alternative approach can be implemented to look into the nature and immediate implications behind the term in question. The suggested approach involves investigating the occurrences of the term proper as opposed to analysing individual buzzwords. The authors hypothise and demonstrate that linguistic context of the term “buzzword”, i.e. the peculiarities of its occurrence alongside different language units and patterns, may provide credible inferences concerning the key properties attached to the term and afford some illuminating perspective on the prevailing attitudes towards it. The study uses titles of research and newspaper articles retrieved from Google Scholar as material for the research due to better representation of the term in titles as opposed to full texts, as well as titles' higher informativity and better descriptive saturation. Apart from the prevailing focus on the immediate linguistic context surrounding the term, the inferences made in this study also stem from the frequency data showing how often a certain feature of buzzwords is being mentioned in the titles. The study showed that while the greatest emphasis is being placed on the field of use and temporal lifecycle of buzzwords, the attitudes towards them can be best of all described by examining the place of the term in oppositions. As illustrated in the paper, these oppositions reflect the idea of the term's inferior status by opposing it to more favourable concepts.

## 1 Introduction

The present paper deals with buzzwords, which are “important-sounding usually technical words or phrases often of little meaning used chiefly to impress laymen” (Merriam-Webster Dictionary, 2019) and that “have become fashionable by being used a lot” (Online Cambridge Dictionary, 2019). Any conversation about buzzwords – or “vogue words”, or “fashion words” – whether it be linguistic scrutiny or general observational review, will in the nature of things be premised on what lies at the heart of rhetoric itself: the bottom line is, it is all about abiding by the laws of the language and sometimes tweaking them – but with due caution – to ultimately produce effective words that will work together in the sake of persuasion and eloquence, be it inspiringly sincere or covertly disingenuous.

Naturally, a study of buzzwords commonly implies doing just this – studying individual language units perceived as buzzwords. Only a little digging will be enough to unearth a long roster of fashionable catchwords with elusive meaning that have emerged within a certain institutional discourse, such as trade, agriculture, information technology, artificial intelligence, education, advertising, etc. Anyone with a minimum level of exposure to the outside world would recognise the intriguing items such as *synergy, leverage, globalisation, development* or *paradigm shift*, although defining them accurately is what usually proves challenging.

As literature review below will show, meticulous examination of individual vogue words is something that has been done extensively and from a variety of perspectives, which has contributed immensely to the research on buzzwords. This includes identifying their functions, tracing their etymology, specifying their meaning, coming up with a precise definition for the fashionable word in question, and even measuring people's ability to predict whether a certain word is going to create a buzz eventually. This line of research with the focus on individual vogue words found in various institutional narratives is covered amply in scientific literature and has provided grounds for specifying the key characteristics of buzzwords: they are chiefly coined within a certain (usually professional) community, they gain wide acceptance by being used a lot, they become fashionable as they grow popular, and their semantics is ambiguous for various reasons. Thus, a typical definition found in academic literature and dictionaries will read as follows:

“A buzzword is a word or expression that has become fashionable in a particular field and is being used a lot by the media while having little or imprecise meaning but sounding impressive to outsiders” (a collective definition compiled using Collins English Dictionary, 2019).

While these key features of buzzwords have been identified through scrupulous examination of individual examples, the present paper poses a question of whether these same key features will come to light if we analyse the use of the term “buzzword” proper. The hypothesis for the study postulates that linguistic context of the term “buzzword” proper, i.e. the peculiarities of its occurrence alongside different language units and patterns, may provide credible inferences concerning the key properties attached to the term and possibly afford some illuminating perspective on the prevailing attitudes towards it. Thus, this study takes up a different approach by basically denouncing any critique of individual fashion words perceived as buzzwords and looking instead into the treatment of the term itself.

Guided by this hypothesis, we aim to single out and analyse the occurrences of the term “buzzword” in order to address the following research question: can the linguistic context of the term proper be indicative of the properties behind it and attitudes towards it? This is an alternative, previously unpractised “shortcut” approach (insomuch as we only deal with the occurrences of the term proper as opposed to analysing various and numerous individual buzzwords) that might be helpful in at least clarifying the meaning behind the term as well as the firmly established attitudes towards it.

## 2 Literature review

The dominant property of a buzzword, and the one stressed most persistently in the literature, is easily deduced from its self-explanatory “buzz” component with expressions like “get a buzz out of something”, or “create a buzz”, or “what's the buzz” coming to mind in association with something exciting or intriguing. In the long run, creating intrigue and drawing attention actually came to serve as the central functions attributed to buzzwords often referred to as “fashion words” (Neuman et al., 2011: 58).

This concept of an attractive “shell” designed to intrigue and grab the attention of the people is widely criticised by scholars. The tendency to “package common-sense ideas under seductive new names” (Cluley, 2013: 34), prioritise “glamorous decorations” over meaning (Cornwall and Brock, 2005: 1043), and basically turn a seemingly serious conversation into a tapestry of exciting catchphrases is frowned upon in numerous studies on buzzwords. The criticism is mostly grounded on the argument suggesting, not without reason, that this kind of intrigue is faultily rooted in the less exciting attributes of ambiguity and mendacity seen as tools for obfuscation hindering genuine communication (Bauman, 2007; Cornwall and Brock 2005; Kilyeni, 2015).

Thus surfaces the controversy surrounding buzzwords that represent abstract notions agreed upon within a linguo-cultural community even though their practical meaning remains a subject of debate, something Gallie (1956) termed “essentially contested concepts”. The contestability, the abstract nature, the vague and euphemistic qualities of buzzwords, however, is what makes them part of vague language often seen as a strategy for achieving goals (Malyuga and McCarthy, 2018: 41) and enables them to assume an array of possible meanings and incompatible interpretations to ultimately become “consensual hurrah-words” (Chandhoke, 2007; Cornwall, 2007: 472; Scoones, 2007).

Admittedly, some scholars try to see beyond the semantic baggage that makes their meaning challenging to decipher. Cunningham and Greene (2013), for example, believe that if a word is capable of invoking the feeling of excitement and helps draw attention to the topic being discussed, it shouldn't be discarded as flawed and treated with suspicion. The authors consider examples such as *bottom line, globalise, incentivise, proactive, robust, synergy* and *value-added*, and encourage using the buzzword if it is “lively and capable of injecting some spunk into a dull sentence” (Cunningham and Greene, 2013: 122).

Quite remarkably, the analogy with fashion has more to it than the on-the-surface idea of an item of clothing, a place or activity being considered stylish or acceptable. Being fashionable (be it in terms of appearance or the language one chooses to use) is not so much an individual trademark, but rather a commitment to identify oneself as part of a community. This aspect of buzzwords prompted scholars to describe them as elements of a shared language (Goodwin, 2014), exclusive code-words (Cornwall, 2007: 472), and linguistic indicators of a key social skill (Richards, 2017).

The importance attached to trendiness and the time-sensitive nature of buzzwords brings to the forefront the temporal aspect of the issue, for “different words buzz at different times and in different contexts” (Cluley, 2013: 37), after which time they lose their popularity and fall into disuse (Neuman et al., 2011: 58). For example, tracking the buzzwords of the American business community, Alderman (2011) draws a prominently visible timeline where *management by objective, synergy* and *vertical integration* were topping the business charts in the 1970s, 1980s and 1990s, respectively.

Buzzwords have been established to originate within a specialist field, coined by experts in this field and, thus, originally, not readily comprehensible outside the professional community (Baker and Sangiamchit, 2019; Kalugina et al., 2019). As the new term gains wider acceptance through continuous use, it gets adopted outside of its original context to be further exploited by a wider public (Karimzad, 2020), and it is this process that sets the scene for the gradual loss or degradation of its meaning. The resulting misappropriation deprives the buzzword of its original semantics to a varying degree, as is the case, for example, with *iterate*, which used to mean “a design process where various elements would progress through sequential steps, to hone in on the optimal solution; and now it means nothing beyond merely describing a stage in a process” (Goodwin, 2014).

Since language is very much associated with power relations, the subject of politics and power in buzzword coinage and dissemination processes has received the spotlight in numerous studies. For example, Arnold (2008) analyses *leverage* only to observe its heavy use in the discussions on the financial crisis, the buzzword essentially acting as an eloquent pledge thrown in to assure maximum investment returns. By the same token, Rist (2007: 486) states that *development* was introduced into the political discourse to be used as “an excuse for enticing “developing countries” to side with one camp or another”, while at the same time remaining vague enough for the politicians to be able to exploit the buzzword for their own benefit (Banguis-Bantawig, 2019). This is something Mjøs et al. (2014) describe as “strategy legitimisation”: the fuzzier the meaning of the term, the more general strategies it can serve.

Also critical to the political agenda is buzzwords’ ability to protect the ideas, initiatives and measures behind them from criticism. The protective mechanism operates on not just the fuzziness of the term alone, but also on its capacity to evoke positive emotions (Mayes and Tao, 2019), trigger favourable connotations, and invite automatic approval, all of which Standing (2007) refers to as the “feel-good factor”. A good case in point is *diversity* which, as shown in Prasad et al. (2011: 714), was statistically proven to be used by managers chiefly to “frame a set of decisions in a way that protected those managers from potential criticism”.

Carrying this rather powerful inventory, buzzwords inevitably make those using them sound authoritative as they help exert control over the dazzled audience. As Cluley (2013: 37) puts it, it is not because buzzwords indicate expert knowledge, but because they help people survive without any of it by essentially looking for the word instead of the answer.

The lack of meaning does not warrant its total absence (Smirnova, 2016): in order to function as a buzzword, a word will still need to have at least a hint of appropriate semantic content (Cairns and Krzywoszynska, 2016), for otherwise it will only leave the audience confused, as opposed to intrigued. Although one of the functions of buzzwords is to simplify a complex field (Mjøs et al., 2014), they must also operate as language units that help discuss existing concepts in new ways, which would be inconceivable should one infest this discussion with the vocabulary bearing zero semantic load. For example, *engagement*, although not quite readily interpreted in terms of the intricate details of its definition, still communicates a recognisable meaning – how much one loves one's job. In fact, Huppke (2015) argues that companies witnessed improvement in productivity ever since the concept of *engagement* was introduced into their professional discourse: somehow, labelling the idea of being invested in your work helped motivate the employees to live up to the new label and the expectations it brought in.

Last but not least, a rather appealing line of research on buzzwords, and the one revealing the key mechanism behind their conception and proliferation, involves searching for the factors that predict which words have the potential to become buzzwords in the future. Thus, Tomonaga et al. (2014) analyse the timespan of individual buzzwords and search for their earliest mentions by bloggers before their peak in popularity to propose a method for evaluating bloggers’ buzzword prediction ability. Pursuing a similar strand of research, various scholars suggested ways to predict what topics would likely lay the foundation for future buzzwords (see Nakajima et al., 2012; Okumura, 2006; Furukawa et al., 2009).

The literature review prepared for this study has revealed opposing views on the expediency of buzzwords with different scholars discussing different dimensions of the concept and its relevance in various contexts. At that, the critique on buzzwords ranges from mildly compromising to overtly rebellious, referring to buzzwords as “toxic” terms (Rist, 2007: 485) and “overused workplace gibberish” (Huppke, 2015), and encouraging getting rid of buzzwords altogether, because “the world would be better without them” (Jay, 2004: 55). The following research on the occurrences of the term “buzzword” will show if the same tendency shall come to light should we apply the proposed “shortcut” approach to studying vogue words.

## 3 Materials and methods

To study the occurrences of the term “buzzword”, we have chosen to contemplate its application in the titles of research and newspaper articles. The focus on titles, as opposed to full texts containing the word “buzzword”, stems from the specificity of both scientific and journalistic texts dealing with buzzwords that tend to almost exclusively focus on individual examples of fashion words. This means that the papers and articles behind the examined titles, while addressing buzzword-related issues, do not actually mention the term itself nearly as much as the specific word they set out to examine, be it *development, empowerment, engagement* or the like. Another argument in favour of titles as research material is their augmented informativity driven by the need to adjust the heading length-wise while still retaining high-priority keywords crucial to the content of the text (see Buxton and Meadows 1977; Soler, 2007). We consider higher informative saturation an exceedingly valuable feature of titles, for it fuels a more prolific use of pre- and post-modifiers that are essential to the present study as we focus on the co-occurring elements accompanying the term “buzzword”. Lastly, both research papers and newspaper articles tend to summarise the key ideas of the narrative synoptically in their headings to attract the readers’ attention (Chen et al., 2015; Bowles and Borden 2000; Ellis, 2001; Saxena, 2006). This prompts the authors to formulate their titles in the most impactful and conclusive way, which, among other things, entails using impactful and conclusive language in their titles.

One of the key methodological premises of the study suggests that a search for the co-occurrences of the term in question within the titles can help illuminate a range of associations related to it and, hence, explain the content of the term for better interpretation purposes. This perspective has been addressed in a number of papers assessing various approaches to term extraction and occurrence evaluation, some of which have been taken into account in this study in developing a relevant research methodology.

For example, Wei et al. (2017) conducted textual topic evaluation analysis based on term co-occurrence through a case study on government work reports and ultimately singled out 12 prevailing topics associated with the term, something the authors called “topic modelling”. This approach is adapted in this study as we look for categories of marked occurrences and label them accordingly later on.

The relevance of immediate co-occurrences is stressed, among others, by Ma et al. (2017), who suggest that co-occurrence distance determines the “weight” of the term, i.e. the tokens (items) or syntactic structures immediately following or preceding the term are the ones that should be considered the most valuable in assessing its ultimate associations. This assumption has also been factored in as we attempt to look for the immediate lexical and syntactic co-occurrences of the term “buzzword” to come up with its comprehensive interpretation.

A search for the most suitable database for material retrieved has uncovered many studies contemplating which academic search systems are suitable for systematic reviews or meta-analyses (see, for example, Gusenbauer and Haddaway, 2019), estimating the difference in referencing found in these systems (see, for example, Anker et al., 2019), or comparing the sizes of academic search engines and bibliographic databases (see, for example, Gusenbauer, 2019). Particularly, the findings offered in these papers estimate that with 389 million records Google Scholar is the most comprehensive academic search engine to date and has a higher citation count than most of its known counterparts.

Since this paper intends to analyse the occurrences of the term “buzzword” in research and newspaper titles, the material for the study had to be collected via an existing database for written research and journalistic content, such as Google Scholar, which was ultimately resorted to as one of the most representative web search engines indexing the full text and metadata of scholarly literature across an array of publishing formats and disciplines. The following principles were adhered to in using this search tool:

1) the search for material was conducted through search queries implemented in two stages to incorporate two main morphological forms of the word in question, i.e. the singular “buzzword” and the plural “buzzwords” in order to ensure better coverage;
2) no filters in the advance search mode, such as specific time period or relevance, were applied for the same reason;
3) the search was conducted in incognito mode to ensure that no previous searches influenced the ultimate results.

Continuous sampling of material retrieved a total of 719 titles. The sample was further grouped per different functional and pragmatic markers, derived and labelled by us, whereby the final volume of the processed occurrences eventually expended as many of the titles incorporated more than one marker of interest. Since the study is not concerned with the content of research presented in the sample and only intends to look into the immediate language units accompanying the term in question within the titles, the sampling method did not support any kind of differentiation content-wise and incorporated titles of newspapers and research articles concerned with various areas of scholarly interest, including internet technology, biology, medicine, politics, language, economics, management, business, education, etc. Apart from the prevailing focus on the immediate linguistic context surrounding the term, the inferences made in this study also stem from the frequency data showing how often a certain feature of buzzwords is being mentioned in the titles.

## 4 Results

The search for the occurrences of the term “buzzword” throughout the sample of 719 examples allowed us to differentiate between 11 categories of marked occurrences labelled as follows: intended purpose (4); positiveness (17); coined term (19); taking action or call to action (29); change in perception, implementation or connotation (50); need for clarification, explanation or definition (54); downgrading and critique (87); implication of a wider scope of meaning, application or significance (123); opposition to a better concept (156); field of use (177); temporal lifecycle (198).

The unmarked occurrences comprised as few as 34 examples which amounts to only 4.7% of the sample. These included neutral titles such as *Intersectionality as Buzzword; Innovation: The History of a Buzzword; Buzzword: Green Fatigue; The Buzzword is “Espirit”* and the like, containing only general informative wordings revealing no marked characterisation or attitudinal disposition whatsoever. In fact, the low percentage of unmarked occurrences may on its own be viewed as a telling argument suggesting that the term “buzzword” proper was rarely free of the superimposed assessments reflected in the immediately approximate language units in the sample.

Grouped in terms of the frequency of occurrence, 6 out of the 11 allocated categories did not exceed the 10% threshold having surfaced up to 54 times. The least represented is the Intended Purpose category with only 4 examples (0.6%) registered in the sample. These were allocated as directly describing the intentions behind introducing or using a buzzword, as in *Three Buzzwords for Maintaining Ethical Hourly Billing*, or *A Buzzword to Enhance Storymaking*.

The next poorly represented category is Positiveness incorporating only 17 examples and amounting to 2.4% of the sample. The co-occurrences in this category included language units either bearing a straightforwardly positive connotation or at least not directly implying any negative meaning, as in *right, worth remembering, big, the biggest, important, well-known, popular, money-making, global, effective, good,* and *favourite*. A single occurrence in the category – *missing* – appeared remarkable as the only one suggesting that a certain crucial buzzword is missing from the professional discourse and needs to be introduced into it: *Fault-Tolerance: Java's Missing Buzzword.*

While individual buzzwords do live up to their name being associated with intrigue and newness, the term proper has also given rise to catchy coinage. The study registered 19 instances of Coined Terms amounting to 2.6% of the sample and including nomenclature such as *buzzword approach, buzzword bingo, buzzword prediction ability* and *buzzword detection*, all referring to a specific concept treated as a subject of scholarly interest.

The Taking Action or Call to Action category incorporated 29 occurrences (4% of the sample) whereby the term “buzzword” was accompanied by a verb or a verbal construction that either referred to a buzzword-related action or straightforwardly encouraged one (in the titles of imperative type). In the former case, the action was rendered through the verbs and verbal constructions such as “retire” (e.g. *Why It's Time to Retire “Disruption”, Silicon Valley's Emptiest Buzzword*), “fill with” (e.g. *Filling a Buzzword with Life*), “make the most of” (e.g. *Making the Most of the Latest Buzzword*), “hype” (e.g. *Hyping the New Media Buzzword*), “avoid” (e.g. *Lawmakers Avoid Buzzwords on Climate Change Bills*), “overcome” (e.g. *Overcoming Buzzwords and Variability Through a Nurse EBP Mentor Program*), “catch” (e.g. *Catching the Buzzwords*), “predict” (e.g. *Finding Prophets in the Blogosphere: Bloggers Who Predicted Buzzwords Before They Became Popular*), turn into (e.g. *Turning Buzzwords into a Process*) and monitor (e.g. *A System Approach to Monitor Buzzwords in Plain English Publications*). In the latter – more vividly emphatic – case, the encouragement to denounce buzzwords appeared exceedingly overt with imperative sentences urging the reader to forget, ban, stop using, say no to, tear down or steer clear of buzzwords. Some of the more over-the-edge instances can also be described as slogans that might just as well be part of an agitation campaign, as in *Death to Buzzwords; Enough with the Buzzwords; Please, No More Buzzwords; Please, Business Instead of Buzzwords; Do not Let IRM Become Another Buzzword*, etc.

The category reflecting the idea of a Change in Perception, Implementation or Connotation (50 examples amounting to 7% of the sample) is represented by the “from – to” pattern suggesting that a buzzword is going through a certain transformation, be it in terms of the meaning attached to it, or its ultimate status within a specific field of use. Some of the more recurrent examples include *From Buzzword to Practice; From a Buzzword to a Definition; From Buzzwords to Strategies; From Buzzword to Reality*, etc.

7.5% of the sample (54 examples) incorporated the occurrences that implied the idea of the Need for Clarification, Explanation or Definition. Apart from the direct nomination of a clarifying action (e.g. *Dispelling the Myths About One of Publishing's Hottest Buzzwords; Sorting Out a Meaning for a Confusing Buzzword; Finding a Way to Define the New Buzzword; Demystifying the Buzzword; Exploring the Meaning of an Internet-Born Digital Marketing Buzzword; Rethinking Buzzwords*, etc.), this category also included the words *guide, dictionary* and *glossary* as recurrent co-occurrences, as in *A Quick Guide to Social Change Buzzwords; A Dictionary of Business Buzzwords; A Buzzword Glossary*. Since these did not actually present guides, dictionaries and glossaries in the conventional sense, the implication was that the study or article was aimed at clarifying and structuring the available information on the buzzword or buzzwords in question.

The next category containing 87 examples and amounting to 12.1% of the sample was labelled as Downgrading and Critique and consisted of occurrences that implied a varying degree of negative connotation bestowed on the term. The category is represented by co-occurrences such as *just, yet another, merely* and *simply* transferring the semantics of the deficient status of a buzzword, as in *just another buzzword, yet another buzzword, merely a trendy buzzword, simply another buzzword*. The same category also incorporated linguistic units accompanying the term in question and transferring the critically-coloured meaning of abundance and abuse (*swarm of buzzwords, beset by buzzwords, too many buzzwords, overhyped, hackneyed, overused, abused buzzword*), semantic insufficiency (*meaningless, empty, ambiguous, fuzzy, confusing buzzword*) and general critique of buzzwords (*ugly, irrelevant, annoying buzzword*).

According to frequency calculations, Implication of a Wider Scope of Meaning, Application or Significance is a yet more voluminous category incorporating 123 examples amounting to 17.1% of the sample. The category is represented exclusively by three recurrent patterns accompanying the term “buzzword”: “more than” (e.g. *Partnership: More Than a Buzzword), “behind” (e.g. Innovation: Behind the Buzzword*), and “beyond” (e.g. *The History of Fileless Malware: Looking Beyond the Buzzword*). This kind of wording suggests that there's something more to a buzzword beyond its on-the-surface attributes so that something bigger can be unearthed if one goes the extra mile and digs a little deeper.

Opposition to a Better Concept incorporated 156 examples amounting to 21.7% of the sample and ranked third among the entire set of allocated categories in terms of the frequency of occurrence. The exemplars included in the category are represented by bipolar oppositions whereby “buzzword” is weighed against a contrastive and more favourable member of the opposition. For example, in *User Experience: Buzzword or New Paradigm*, “buzzword” obviously loses the battle when opposed to “new paradigm” as it is being automatically set aside as something “not new” and “not paradigmatic”. In *Ontology Simplification: New Buzzword or Real Need*, the same reasoning applies as the title unflatteringly suggests to contrast “buzzword” with something “real” and actually “needed”.

Field of Use appeared as one of the most representative categories having incorporated 177 examples amounting to 24.6% of the sample. The category included co-occurrences referring to vogue words reserved for a specific institution (as in *management, business, marketing, retail, internet* buzzword) or community (as in *experts’, online, office, student* buzzword).

The hottest on the list is the Temporal Lifecycle category referring to the endurability of a buzzword from conception to decay (as in *birth of a buzzword, half-life of a buzzword, an old buzzword is back, a buzzword in the making, no longer a buzzword, buzzword mutation*) and the general temporal and trend-dependent characteristics as the key features of buzzwords (as in *today's buzzword, former buzzword, this season's buzzword, this year's buzzword, buzzword of 2016, 1970s buzzword, flavour-of-the-month buzzword, buzzword of yesteryear, new, old, next, latest, current, hottest buzzword*). The category is represented by 198 examples amounting to 27.5% of the sample.

As can be seen from the presentation of study results, the applied route of research involving the analysis of linguistic units and patterns accompanying the term “buzzword” in the given sample did not reveal a large number of intricate features that could otherwise be singled out by investigating individual vogue words. We didn't, for example, deduce any recurrent overt references to the political agenda, or avoidance of criticism, or the sense of authority, or migration of buzzwords from field-specific to broad colloquial discourse. The lack of such references in the ultimate data breakdown, however, attests not to the deficiency of the proposed approach, but rather to the lesser significance of these particular references. In this vein, being oriented towards an explicit listing of most recurrently credited priority features of buzzwords and their frequency-based rating, the suggested line of research can be viewed as a “shortcut approach” to isolating and ranking the key attributes of buzzwords, which can be put to good use to help us clarify the definition of the term.

## 5 Discussion

At the very onset of the research, the low percentage (4.7%) of neutral titles containing no marked linguistic units to characterise the term “buzzword” was in itself an indicator signalling the feasibility of the adopted approach: the fact that the term rarely appeared as a stand-alone item, devoid of any adjoining attributive descriptive language tokens and the attitudinal appraisal that came along with them, verified the controversial nature of the term as a far-from-neutral concept. While the nomenclature of the allocated categories and the functional interpretation of their content is very much consistent with the observations presented in the literature review, frequency calculations coupled with the analysis of recurrent co-occurrences are what might be considered a staging ground for valuable inferences.

Thus, for example, the lack of a straightforward purpose behind buzzwords can be deduced from the poorly represented category of Intended Purpose that incorporates as few as 4 examples referencing the purposeful application of buzzwords, as in *Yet Another Buzzword to Hide our Confusion,* or *The Buzzword to Ensure Occupational Safety and Health.*

The 2.4% of occurrences referring to the positive features of buzzwords alongside the 21.1% of occurrences transferring the meaning of downgrading and critique, present statistical data reflecting the prevailing attitudes towards the notion of vogue words. At that, the attitudinal aspect is also clearly reflected in the language chosen to express the idea of positive assessment and criticism, which differs strikingly in terms of the register being used, with positive features explicated through commonplace adjectives such as *right, important, well-known, good*, etc., and criticism communicated via much more toxic descriptive elements such as *hackneyed, ugly* and *annoying.*

The action-related occurrences that either referred to some buzzword-related action or explicitly encouraged one are valuable insofar as the immediate verbal context accompanying the term “buzzword” in the titles can also vividly illuminate the nature of activity most commonly linked to it. Seeing that the co-occurring verbs and verbal constructions in this category include *retire, hype, avoid, ban, overcome, predict, monitor, forget, stop using, say no to, steer clear of, tear down,* etc., the content of the category Taking Action or Call to Action attests to the so far consistent idea of disapproval. Some of the more appellative examples of the call to action include *Message to New Government: Please Ban Buzzwords; Just Say No to Buzzwords; Make “Trust” a Cornerstone, not a Buzzword; Steer Clear of Buzzwords; Forget All the Expert's Buzzwords,* etc.

Yet despite – or maybe due to – this controversial nature of buzzwords, the study revealed that as a subject of scholarly interest they might just be on the fast track of becoming a fruitful source for coinage. The topic of buzzwords generated coined terms such as *buzzword approach* (forming of policies by privileging rhetoric over reality), *buzzword bingo* (a bingo-style game where participants prepare bingo cards with buzzwords and tick them off when they are uttered during an event, such as a meeting or speech), *buzzword prediction ability* (an ability to predict whether a certain word will become a buzzword in the future) and *buzzword detection* (spotting of “buzzworthy” language units functioning in different types of discourse).

Change in Perception, Implementation or Connotation, although relatively insignificant with only 50 examples (7% of the sample) fitting into its semantic scope, triggered some valuable inferences which was mostly due the consistently used language pattern in the corresponding titles. As the “from – to” construction signified the process of transformation, whereby the term “buzzword” was marked as the starting point for the change (Marker 1), the following juxtaposition was effectuated to compare it to the ultimate point of “destination” (Marker 2) in this kind of transitioning, as in:

• (From) Buzzword (to) Reality
• (From) Buzzword (to) Practice
• (From) Buzzword (to) Best Practice
• (From) Buzzword (to) Strategy
• (From) Buzzword (to) a Definition
• (From) Buzzword (to) Business Strategy
• (From) Buzzword (to) Action
• (From) Old Buzzword (to) New Praxis
• (From) Buzzword (to) Theory
• (From) Buzzword (to) Non-negotiable Limits
• (From) Buzzword (to) Value Creation
• (From) Buzzword (to) Real Responsibility
• (From) Buzzword (to) A Useful Concept
• (From) Global Buzzword (to) Specific Solution
• (From) Buzzword (to) A Life Sciences Approach
• (From) Buzzword (to) Research Priority
• (From) Marketing Buzzword (to) Market Relevance
• (From) Buzzword (to) Strategic Imperative
• (From) Buzzword (to) Business Practice
• (From) Buzzword (to) Payoff
• (From) Buzzword (to) Instrument
• (From) Buzzword (to) Managerial Tool
• (From) Buzzword (to) Necessity
• (From) Buzzword (to) Weapon
• (From) Buzzword (to) Critical Psychology
• (From) Buzzword (to) Managerial Tool
• (From) Buzzword (to) Megatrend
• (From) Buzzword (to) Implementation of Learning Analytics
• (From) Buzzword (to) A Social Construction of Competency
• (From) Buzzword (to) Pro-Environmental Behaviour Change
• (From) Buzzword (to) Mainstream Web Reality

Thus, the retrieved examples either opposed the term to something “real”, “new”, “better”, or “useful”, or encouraged to see it as a thing of the past that now has to give way to “behaviour change”, “construction of competency”, “research priority”, and “value creation”.

A yet more representative category amounting to 7.5% of the sample referred to the need to clarify, explain or provide a clearer definition of a buzzword. The choice of wording in this case was very much straightforward, as in *dispelling the myths about/understanding/rethinking/unravelling/sorting out a meaning for/defining/finding a way to define/demystifying/exploring/mapping out buzzwords; buzzwords explained/defined*, etc. Some titles transferred the same meaning through declarative statements thus making the idea stand out a little bit more, as in *“Analytics” Buzzword Needs Careful Definition,* or *Globalization Is a Buzzword that Has No Precise Definition,* or *Riding the Waves of “Web 2.0”: More Than a Buzzword, But Still Not Easily Defined*. The same is true for titles in this category formulated in the interrogative from, as in *“Student Experience” Is the New Buzzword: But What Does It Mean?; Putting the Science Back in C2: What Do the Buzzwords Really Mean?; “Agency” is the Buzzword, But Where Does It Work?,* etc.

A much more generous proportion of examples referred to the implication of buzzwords' wider scope of meaning, application or significance with the category represented by the pattern “more than” and lexical units “beyond” and “behind”, as in *Asset Management is Much More than an Annoying Buzzword; Omnichannel: More Than a Digital Transformation Buzzword; Beyond the Buzzword: The Three Meanings of “Grand Strategy”; Behind the Buzzword: Employability,* etc. Although 10 out of 123 examples were formulated as questions, these were still included as bearing the same implication, as in *Software Reuse: What's Behind the Buzzword?; Turnaround Management: More Than a Buzzword?; Is MOOC More Than Just a Buzzword?*

The largest three categories were examined more scrupulously as the most representative. Consistently with the observations provided in the literature review, the categories of the Field of Use and Temporal Lifecycle were the top two categories providing for 24.6% and 27.5% of the sample, respectively. The former was represented by references to institutions (as in *financial buzzword*) or communities using the vogue word (as in *urban buzzword*), while the latter made use of versatile linguistic context to indicate time-dependent properties of the term, as in *half-life of a buzzword, today's buzzword, this year's buzzword, buzzword of the decade, buzzword of a post-PC era, new, latest, next, old buzzword,* etc.

However, it was the category that referenced opposition to a better concept (21.7% of the sample) that proved the most illuminating. Similar to the titles that referred to the process of change in perception, implementation or connotation of buzzwords, the opposition category highlighted the idea that the term is all too often contrasted with a better, more promising or viable alternative. The buzzword was most recurrently opposed to reality, relevant concept, new paradigm, innovation, best practice and breakthrough, as in *Buzzword or Reality; Buzzword or Relevant Concept; Buzzword or New Paradigm*, etc.

## 6 Conclusion

The paper proposed and applied an alternative approach to studying buzzwords, whereby the occurrences of the term within a clearly defined sample were analysed instead of traditional examination of individual buzzwords. While the “shortcut” approach did not allow to track down the intricate features of buzzwords – such as their expediency in avoiding criticism, instilling a sense of authority or bolstering political agenda – it did allow to single out the same key features as those mentioned in the texts dealing with individual buzzwords. It also proved useful in listing the most recurrently credited features of buzzwords as per their frequency-based rating. The study showed that while the greatest emphasis is being placed on the field of use and temporal lifecycle of buzzwords, the attitudes towards them can be best of all described by examining the place of the term in oppositions. As illustrated in the paper, these oppositions reflect the idea of the term's inferior status by opposing it to more favourable concepts. At that, the tendencies in representing buzzwords' less frequently credited features have also attested to the general trend whereby the term and the notions behind it are associated with a far-fetched, opportune and often meaningless rhetoric having little to do with reality.

This study contrasts with much of the work in the literature review in that it analysed buzzwords in a limited context, typically a phrase or short sentence, and drew inferences as to their function from their syntactical role or collocational characteristics. The advantage of this approach is that it allows a large quantity of data to be analysed across a wide range of contexts so that the results are representative of the buzzword phenomenon. The majority of studies have taken a narrower and deeper direction, typically tracing the function of a specific buzzword in one text type at the level of discourse. This approach is of course no less valid but the present study represents a meta-analysis of buzzwords which is not dependent on their individual usage.

## Declarations

### Author contribution statement

Elena N. Malyuga & Wayne Rimmer: Conceived and designed the experiments; Performed the experiments; Analyzed and interpreted the data; Contributed reagents, materials, analysis tools or data; Wrote the paper.

### Funding statement

This research did not receive any specific grant from funding agencies in the public, commercial, or not-for-profit sectors.

### Data availability statement

Data included in article/supplementary material/referenced in article.

### Declaration of interests statement

The authors declare no conflict of interest.

### Additional information

No additional information is available for this paper.
